# Non-Random Variability in Functional Composition of Coral Reef Fish Communities along an Environmental Gradient

**DOI:** 10.1371/journal.pone.0154014

**Published:** 2016-04-21

**Authors:** Jeremiah G. Plass-Johnson, Marc H. Taylor, Aidah A. A. Husain, Mirta C. Teichberg, Sebastian C. A. Ferse

**Affiliations:** 1 Department of Ecology, Leibniz Centre for Tropical Marine Ecology (ZMT), Bremen, Germany; 2 Faculty of Biology and Chemistry (FB2), University of Bremen, Bremen, Germany; 3 Faculty of Sciences, University of Nice Sophia-Antipolis, Ecology of Marine Ecosystems and Responses to Stress (ECOMERS), Nice, France; 4 Department of Theoretical Ecology and Modelling, Leibniz Centre for Tropical Marine Ecology (ZMT), Bremen, Germany; 5 Thünen Institute of Sea Fisheries, Hamburg, Germany; 6 Center for Marine, Coastal and Small Islands Research, Hasanuddin University, Makassar, Indonesia; 7 Department of Geography, University of Hawaii Mānoa, Honolulu, Hawaii, United States of America; Biodiversity Research Center, Academia Sinica, TAIWAN

## Abstract

Changes in the coral reef complex can affect predator-prey relationships, resource availability and niche utilisation in the associated fish community, which may be reflected in decreased stability of the functional traits present in a community. This is because particular traits may be favoured by a changing environment, or by habitat degradation. Furthermore, other traits can be selected against because degradation can relax the association between fishes and benthic habitat. We characterised six important ecological traits for fish species occurring at seven sites across a disturbed coral reef archipelago in Indonesia, where reefs have been exposed to eutrophication and destructive fishing practices for decades. Functional diversity was assessed using two complementary indices (FRic and RaoQ) and correlated to important environmental factors (live coral cover and rugosity, representing local reef health, and distance from shore, representing a cross-shelf environmental gradient). Indices were examined for both a change in their mean, as well as temporal (short-term; hours) and spatial (cross-shelf) variability, to assess whether fish-habitat association became relaxed along with habitat degradation. Furthermore, variability in individual traits was examined to identify the traits that are most affected by habitat change. Increases in the general reef health indicators, live coral cover and rugosity (correlated with distance from the mainland), were associated with decreases in the variability of functional diversity and with community-level changes in the abundance of several traits (notably home range size, maximum length, microalgae, detritus and small invertebrate feeding and reproductive turnover). A decrease in coral cover increased variability of RaoQ while rugosity and distance both inversely affected variability of FRic; however, averages for these indices did not reveal patterns associated with the environment. These results suggest that increased degradation of coral reefs is associated with increased variability in fish community functional composition resulting from selective impacts on specific traits, thereby affecting the functional response of these communities to increasing perturbations.

## Introduction

Coral reefs are among the most diverse ecological systems in the world due to their complex biogenic composition [1]. Coral reef community composition is a product of interactions among physical (e.g. structural complexity) and biological (e.g. live coral cover and/or benthic diversity) characteristics, creating high structural heterogeneity and providing habitat for many life forms. Most coral reef fishes are dependent on coral reef habitat for shelter and food resources, implying that the structural and biological components of the habitat exert an influence on the composition of the associated fish community. Accordingly, most studies find positive correlations between structural complexity and fish abundance [2], number of species [3,4], biomass [5] and species diversity [6,7].

Coral reefs can experience ecological restructuring in response to anthropogenic disturbances such as changing water chemistry and resource extraction [8]. Resource use and habitat modification can be spatially and temporally dynamic, whereby differing exposure to disturbance can affect ecological processes as movement patterns, competition, predation and recruitment [9]. Trait-specific responses to habitat modification are apparent because species with traits that favour the changed environment are able to become more abundant than prior to the impact. As such, disturbances act as filters on communities, whereby certain species or functions decline in abundance due to environmental constraints (niche filtering; [10]) but species with traits that allow access to new habitat provisions are able to persist or establish themselves.

Coral reef fishes are not only highly diverse taxonomically, but also with a broad range of morphological, biological and behavioural traits [11]. This diversity of traits allows for resource partitioning within this highly speciose group [12]. Conversely, the homogenisation of resources through disturbances can result in communities becoming depauperate in ecological functions and/or species diversity [13]. Traits have been linked to ecosystem functions [13–16] and because of this, trait-based approaches to understanding ecosystem processes have been developed in recent years to help identify mechanisms of community assembly [17–19] and to understand observed patterns of species coexistence [20,21]. For coral reef fish assemblages, trait-based functional analyses have revealed the importance of rare species [22], functional hot-spots [23] and areas of functional loss [24]. These studies have helped describe patterns of functional distribution, and ecological rules that govern assemblages over large spatial scales; however, local disturbances are increasingly recognised as drivers of coral reef communities [25], with the emergence of large-scale patterns as a result of local processes [26].

Localised disturbances, such as point sources of nutrient input, result in spatial gradients that affect coral reefs at different levels. Coral reefs around point sources of pollution may display locally reduced coral biodiversity, low coral recruitment, low skeletal density, high rates of bioerosion, a transition from coral-dominated communities to those dominated by non-reef building organisms and an overall loss of habitat complexity [27,28]. The loss of habitat diversity reduces niche availability for the associated fish assemblages [29]. Conversely, less impacted sites may have higher biological diversity, offering greater resources for niche diversification [30]. Furthermore, disturbance can increase habitat fragmentation, altering niche use for some species, and increasing the presence of opportunistic, less trophically-specialised species.As resources become scarce in degraded habitat patches, the species assemblage will be comprised of transients, traversing patches in search of the resources on which they depend. The fragmentation of habitat and shift to more mobile species may result in reduced stability of the community composition [31]

Increased variability in taxonomic composition and/or abundances of fish fauna has been noted as a response to disturbance [6,32,33]. On coral reefs, disturbance can have deleterious effects on live coral communities with subsequent negative influence on structural complexity. Here, it might be assumed that the responses of the associated fish community may be trait-specific, with reduction or loss of particular traits [34,35] closely associated with live coral and structural complexity, and additionally with resultant changes in abundances of fishes displaying those traits. Furthermore, in a degraded habitat, the occurrence of specific traits may become more stochastic as a result of the presence of vagile species whose traits are not closely linked to the local habitat [31].

While responses of fish communities to localised habitat degradation have been assessed in terms of abundance, biomass, size structure and species richness, our understanding of the extent to which the functional composition of fish communities varies in response to localised reef degradation remains limited. Environmental gradients across a number of distinct reefs allow for the identification of how and which traits are most suited to utilise the immediate environment. Furthermore, a change in the variability in fish traits or fish community functional composition along environmental gradients may act as potential indicators of environmental stress.

While few studies have linked functional loss and functional change of reef fishes to habitat degradation, no study has attempted to identify the specific mechanisms of trait-based functional variability and loss in these assemblages. Results from previous studies on the effects of loss of structural complexity and live coral cover on fish community composition suggest a shift towards larger, less site-attached individuals with larger home ranges at degraded sites, potentially leading to more variability in the species and functions present at a particular point in time; however, this has yet to be tested empirically. Because a changing environment may not equate to net loss of species and/or traits, measuring variability in the composition of traits that are indicative of environmental impact may better reflect the effect of a changing environment on the fish assemblage. This leads to the question, does a healthy environment host a higher number of functions with a higher stability in the functional composition; or conversely, does a degraded habitat result in a community with a reduced and less stable trait composition? This study, therefore, goes beyond traditional taxonomic-based evaluation of habitat degradation, and uses a coral reef archipelago in Indonesia characterised by varying environmental conditions to investigate localised spatial changes in the trait-based functioning of coral reef fish assemblages. The Spermonde Archipelago of Southwest Sulawesi has been identified as a coral reef system displaying varying benthic conditions [36] due to impacts from high coastal population density, industrialisation and marine resource use [37,38]. On-shore to off-shore patterns of live coral cover have been associated with distance from the coastal city of Makassar [37,39], which is reflected in a nutrient gradient [37,40].

We investigated the importance of two key parameters of coral reef health, live coral cover and benthic rugosity, in relation to the functional assembly of the associated fish communities along the spatial gradient. Through a null model approach, we use both the mean of functional indices and the variability of functional composition, along with specific responses of traits, to detect non-random processes affecting coral reef fish community assembly. We hypothesized that reductions in reef health would be reflected in higher variability of the functional composition of the fish community. Furthermore, we predicted that variability in community composition would be driven by traits associated with fish size and (lack of) dependence on live corals as a resource. Lastly, given the importance of identifying habitat loss in coral reef systems, changes in trait-based indices are discussed for the detection of anthropogenic effects.

## Methods and Materials

### Ethical statement

This research was completed in Indonesian waters in accordance with permits issued by the Indonesian Ministry of Science and Technology (Kementerian Riset dan Teknologi; permit number: 352/SIP/FRP/SM/IX/2012). All work was completed in accordance with the code of conduct for animal ethics of the Leibniz Center for Tropical Marine Ecology, Germany, and the University of Hasannudin, Indonesia. All data collection was through observation and no animal, water or substrate materials were collected.

### Study sites and sampling protocol

**Fig 1:**
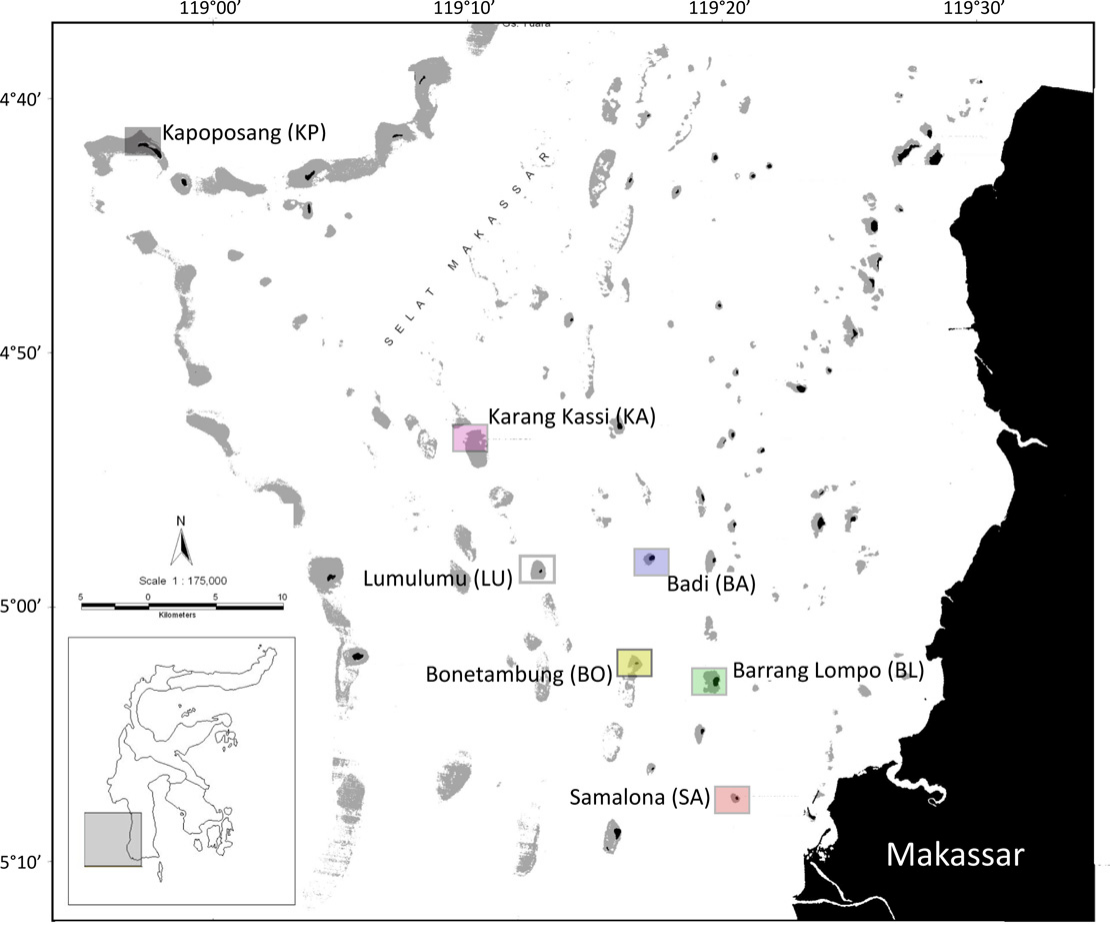
Map of the Spermonde Archipelago. Dashed squares indicate the sampling sites and the in-text acronym is given in parentheses. Colours correlate with subsequent figures. Image modified from Nasa’s Landsat ETM+.

Sampling occurred between the 26^th^ of September and the 2^nd^ of October, 2013, on seven islands of the Spermonde Archipelago, Indonesia, along a transect of increasing distance from the city of Makassar (Fig 1). Makassar is populated by 1.4 million people, and the near-shore islands are affected by effluents from the city’s harbour, sediments, aquaculture outflow and wastewater from the fluvial discharge of the nearby rivers [41]. Samalona (SA; 05°07’S, 119°20’E, 7 km distance from the coast at Makassar) was the site closest to the mainland, followed by Barrang Lompo (BL; 05°02’S, 119°19’E, 11 km distance), Bonetambung (BO; 05°01’S, 119°16’E, 14 km distance), Badi (BA; 04°57’S, 119°16’E, 19 km distance), Lumulumu (LU; 04°58’S, 119°12’E, 22 km distance), Karang Kassi (KA; 04°53’S, 119°09’E, 27 km distance) and Kapoposang (KP; 04°41’S, 118°57’E, 55 km distance; Fig 1).

Sampling sites were standardised at the northwest corner of each island, except for KP. The western side of the islands generally features a well-developed, carbonate fore-reef and a sandy back-reef and flat. The reef crest is shallow (~3 m) and the slope reaches down to 15 m. The last study site, KP, is located on the outer shelf wall of the archipelago and is exposed to deep oceanic waters, contributing to well-developed coral reefs and high biodiversity. Work at KP was conducted at the northeast side of the island at the edge of the carbonate shelf, which was more consistent with the environmental conditions at the other sites.

Fish and benthic surveys were conducted along three 50 m transects at each site. Transects were standardised at 2 m below the reef crest, which for each site fell between 4 and 5 m depth at low tide. Transects were separated by 5 m, and the beginning and end were marked with steel rebar to provide attachment points for the transect tapes. Sampling was conducted across a limited time period to standardise environmental conditions, which may affect fish community patterns among sites. Thus, sampling was timed to occur during the neap tide cycle and during the dry season to ensure consistent conditions for all sampling days.

At each site, the three transects were surveyed three times, by the same observer (JPJ), throughout the day to account for instantaneous variability in fish community composition [42]. The first set was started at the same time of day (8:30 am ± 15 min) at each site, and each transect required approximately 45 min. Surveys were started 30 min after the transect tapes were laid to allow fishes to become acclimated to the presence of the tape, and 30 min were allocated between sets of transects. Surveys by a diver can affect fish behaviour where some species are attracted while some are deterred. Nonetheless, underwater visual surveys are considered an adequate method [43] to describe fish communities. However, the over- or under-representation of some species would affect functional indices and our interpretation of the results tries to account for this. The number and species of all adult fishes >3 cm were recorded within a 5 m wide belt along each 50 m transect. Individuals of the cryptic families, Gobiidae and those in the Blenniidae were not recorded.

### Environmental variables

Live coral cover was quantified at each island with 25 benthic photographic quadrats per 50 m transect. At every second metre, photographs were taken at a standardised height (1 m) above the substratum. On each photograph, ten randomised points were analysed for live hard coral (not displaying necrosis) with Coral Point Count with Excel extensions (CPCE; [44]). Rugosity was assessed with the linear distance-fitted chain method [45]. The chain length used was 20 m, and measurement was conducted once per transect, starting at the first 10 m point.

### Life history traits of fishes

Our choice of traits was similar to those used in recent studies exploring change in functional diversity (FD) of coral reef fishes across large spatial scales [22–24] and for which data are widely available (FishBase, http://www.fishbase.org) (Table 1). They are a combination of morphological, trophic, reproductive and behavioural traits which can be affected by environmental disturbances, leading to changes in species and functional composition (reviewed in [18]). Moreover, the use of combined life-history traits has the potential to better elucidate the effect of disturbance processes on communities [17,18] because disturbances can act on differing sets of traits. Although a direct comparison of fish traits and their ecological importance is not available in the literature, traits were given a weighting, from 1 to 3, based on their ecological importance as interpreted from the literature (Table 1). This weighting accounted for the fact that fish play a highly important role in ecosystem functioning through the regulation of food webs and nutrient cycling [46–48], and that this is directly related to body size [49]. Trophic and size traits were given the highest weight as they also provide a direct biological indication of how species interact with each other and the habitat. Behavioural traits were weighted intermediately because they can change depending on social structure, and depth distribution was given the least weighting because it is limited at all reefs (Table 1). To best capture the trophic role of a species, the trophic description included its two most common foods. Ontogenetic changes of trait values in species were not considered because information on adults is more widely available.

**Table 1:** Life history traits used to characterise a species’ function including weight (in parentheses), definition and values.

Trait	Definition and functional significance	Values
Dietary group (3)	Fish species were described by their two most important dietary constituents. Diet acts as a broad descriptor of ecological interaction, and also, it is a proxy for susceptibility to predation [46–48,50,51].	DG_CO_: Sessile invertebrates (corals and sponges; DG_C1_: Small benthic invertebrates; DG_C2_: Large benthic invertebrates; DG_D_: Detritus; DG_H1_: Macro-algae and seagrass; DG_H2_ Micro-algae and cyanobacteria; DG_P_: Fish or nekton; DG_Z_: Zooplankton
Home range (2)	Species were grouped into three categories based on their normal movements. Sedentary would be exemplified by fishes restricted to relatively small areas (0–5 m^2^); Mobile indicates movement in the 100s m; Wide indicates movement across reefs. Home rage may be an indicator of foraging method along with predation risk and prey defence [24,51]	HR_S_: Sedentary; HR_M_: Mobile; HR_W_: Wide ranging
Schooling behaviour (2)	Schooling is defined according to the normal behaviour of adults of the species. Schooling behaviour may be an indication of susceptibility to predation [51] and of foraging method [52].	SB_A_: Solitary; SB_B_: Paired; SB_C_: Small, loosely aggregated schools; SB_D_: Medium size schools; SB_E_: Large schools
Reproductive turnover (2)	High: Less than 15 months; Medium: Between 1.4 and 4.4 years; Low: 4.5 to 14 years. Reproductive turnover may indicate the capacity for adaptation to quickly changing environments [53].	T_H_: High; T_M_: Medium; T_L_: Low
Depth range (1)	The range of depths which an individual species can transverse, and may indicate susceptibility to fishing [54].	DR: Depth in metres
Max length (3)	Maximum length that a species can potentially reach. Max length is linked to metabolic rate and secondary production [24,48,51]	L_m_: Length in cm

### Measuring observed and expected functional diversity

We constructed a species × species functional distance matrix from a species × trait matrix of all species observed using Gower’s distance [55]. Rao’s quadratic entropy (RaoQ) [56] was calculated by relating the species × species distance matrix to the species abundances. RaoQ reflects changes in the abundance-weighted sum of pairwise functional distance between functional entities and allows for the evaluation of functional diversity within a community [57]. Alternatively, FRic measures the volume of multidimensional functional space occupied by the community [18]. In practical terms, FRic will become greater with the presence of more traits, while RaoQ measures the differences among functional entities within a community. For FRic, we followed the convex hull method proposed by [58]. For further descriptions of the calculation of FRic and RaoQ, see Botta-Dukat [56], Villéger et al. [58], Laliberté and Legendre [59]. These two indices were chosen because they display adequate power to test ecological constraints across environmental gradients [18,19].

To assess the extent to which the observed patterns in indices were linked to site-specific factors structuring community assembly, rather than arising by chance from random assembly of the communities, observed indices were compared to null model results. The null model effectively removes any link between environmental variables and species and their associated traits. Following Gotelli and McCabe [60], the standardized effect size (SES) of observed RaoQ and FRic, which converts the indices to an effective number of functions, was calculated based on the distribution of null model indices:

$$\mathrm{SES}=\left(I_{\mathrm{obs}}–I_{\mathrm{sim}}\right)/\sigma_{\mathrm{sim}},$$

where *I*_obs_ is the observed index, and *I*_sim_ and σ_sim_ are the mean and the standard deviation of the simulated null model indices, respectively, giving a SES for both RaoQ (SESRaoQ) and FRic (SESFRic). In order to calculate functional diversity indices for the null model, the community matrix of observed occurrence frequencies, sample (rows) × species (columns), was permutated 999 times, while the trait matrix was maintained in its original configuration. Null models were generated using the "independent swap" algorithm of Gotelli [61], which randomizes species' occurrence frequencies by sample (i.e. values within each column), but is constrained to only accept permutations that maintain species richness across samples. Although the independent swap null model is designed to maintain patterns of sample richness, sample diversity and abundance are also correlated to some degree. An example of the null model procedure is available as an *R* script from the repository (DOI: 10.6084/m9.figshare.2009331).

### Relationship between functional diversity and the environment

As species richness between replicate counts on individual transects differed due to instantaneous variability in abundance [42] (thus representing the range of possible numbers of species and traits at a transect), the mean functional diversity indices per transect were derived by calculating the SES values for each of the three replicate counts against all iterations for that transect pooled together (3 x 1000). The average of all nine values per island was used as the average index for that island, thus accounting for both temporal and spatial variability at the island level. Variance was calculated by deriving transect-specific SES values for each of the replicate samples (based on 999 permutations), then obtaining a standard deviation (SD) associated with one transect across the three samplings. This created three SDs per site. Mean and variance of both indices were plotted against environmental conditions to check for associations.

Relationships between the SD of the SES indices and the environmental covariates were explored with multiple linear and non-linear models. Collinearity in the covariates was checked with a correlation coefficient, using a threshold of 0.8, and the variance inflation factor (VIF) with a threshold of 3. Coral cover and rugosity were both positively correlated with distance from the mainland (S3 Fig). However, the correlation coefficient was never greater than 0.8 (S1 Fig) nor were the VIFs greater than three (coral cover = 1.4, rugosity = 1.6, and distance from shore = 1.8). These results meant that all covariates were considered for calculating relationships with SESFRic and SESRaoQ. Generalised linear models (GLM) and generalised additive models (GAM) with low rank isotropic smoothers and a gamma distribution were fitted to the full set of covariates, and selection of best sub-models was based on a minimisation of the Akaike Information Criterion (AIC). Model validation was completed via residual plots. To explore the effects of trait weighting on the models, the analyses were repeated without weighting, and a further sensitivity analysis was carried out to test the relative influence of each trait on the observed models. Methods for these analyses can be found in the supplemental material (S1 and S2 Figs).

For all transects, community-level weighted mean values (CWM) were calculated for each trait as the mean trait value weighted by the relative abundance of the species. Mean CWM values per transect were analysed for their responses in relation to the environmental variables. In addition, variability for individual trait CWM was calculated as SD across the three samples of one transect. Linear regression was then applied to see which trait means and variability were most closely linked to the environmental variables. KP was removed from the trait-distance relationship because its inclusion resulted in non-linear relationships. The removal of KP allowed distance-trait relationships among the other six sites to be analysed with linear techniques. The linear results of the first six sites helped to elucidate strong patterns associated with distance from shore, while most KP values were similar to values observed at LU and KA. To identify trait-based associations among species, the first three axes of the principal coordinate analysis (PCoA; applied to the species × species functional distance matrix for calculation of SESFRic) were plotted against each other along with vectors indicating the influence of independent traits. The 15 most abundant species were identified to show how they were related in trait space.

Gower’s distance, Rao’s quadratic entropy, and CWM were calculated with the ‘FD’ package [59], the independent swap null model was introduced with the ‘picante’ package [62], and non-linear models were created with the ‘mgcv’ package [63] in R statistical software [64].

## Results

A total of 31,725 individual fishes were counted, belonging to 144 different species. The species richness was lowest at BL (17.6 ± 1.4 250m^-2^) and BO (15.4 ± 2.1 250m^-2^), and highest at BA (31.3 ± 1.2 250m^-2^), KA (29.6 ± 1.4 250m^-2^) and KP (29 ± 1.7 250m^-2^). Average species abundance was twice as high at BA as at the next site (KA), and the lowest abundances were observed at BL. Despite being the farthest from Makassar, KP had relatively low species abundances (S1 Table).

**Fig 2:**
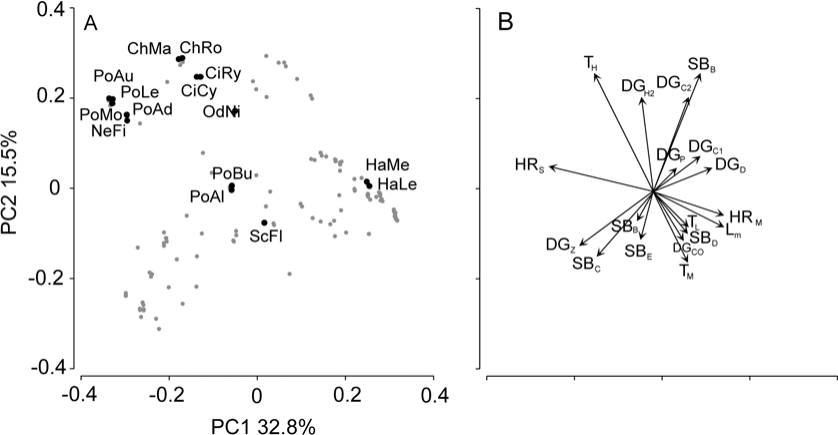
**Biplot of A) first and second PCs of principal coordinate analysis conducted on fish species and traits and B) Vectors identifying traits.** These axes were then used to calculate SESFRic. The fifteen most abundant species have been labelled and are: *Chromis margaritifer* (ChMa), *Chrysiptera rollandi* (ChRo), *Cirrhilabrus cyanopleura* (CiCy), *C*. *ryukyuensis* (CiRy), *Halichoeres leucurus* (HaLe), *H*. *melanurus* (HaMe), *Neopomacentrus filamentosus* (NeFi), *Odonus niger* (OdNi), *Pomacentrus adelus* (PoAd), *P*. *alexanderae* (PoAl), *P*. *aurifrons* (PoAu), *P*. *burroughi* (PoBu), *P*. *lepidogenys* (PoLe), *P*. *moluccensis* (PoMo), *Scarus flavipectoralis* (ScFl). B) Vectors identifying traits (see Table 1 for trait labels) that discriminate species along PCoA axes in plot A.

**Fig 3:**
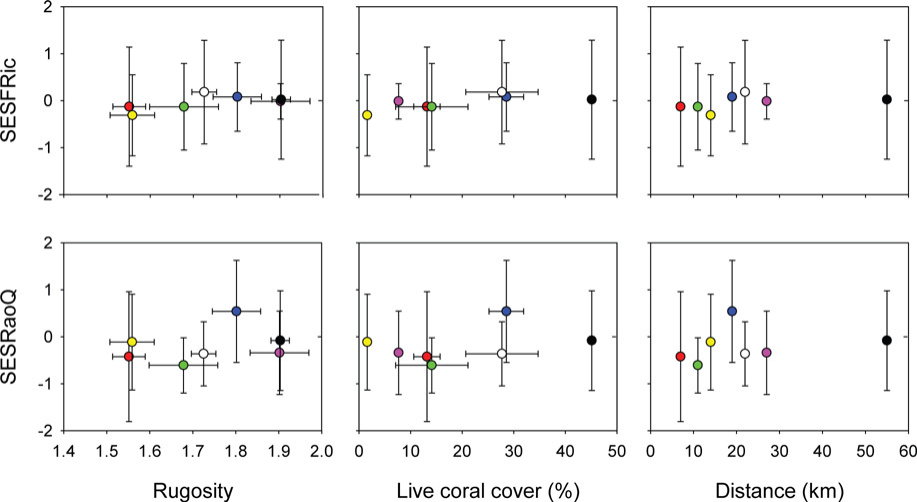
Mean index-environment relationship. The dashed line at 0 of the index represents the value expected under random community assembly, while mean values (±SE) above or below indicate a deviation from the expected as calculated by the standard effect size (SES). Colours represent sampling sites and correlate with Fig 1: red = SA, green = BL, yellow = BO, blue = BA, white = LU, pink = KA, and black = KP.

Among the three environmental factors, live coral was related to the most traits. This included negative relationships with maximum length (L_m_), all home range sizes (HR_S_, HR_M_, HR_L_), dietary groups DG_C1_ (small invertebrates), DG_H2_ (microalgae / cyanobacteria feeding), DG_D_ (detritus feeding), all reproductive turnovers (T_L_, T_M_, T_H_) and large schools (SB_E_). Rugosity was negatively related to small invertebrate feeders (DG_C1_), maximum length (L_m_) and small home ranges (HR_S_). Distance was only (negatively) related to small invertebrate feeders (DG_C1_). None of the traits were associated with rugosity or distance from the mainland alone (Table 2). The correlation between coral cover and home range decreased with home range size, and the correlation with reproductive turnover similarly was strongest for high turnover. Variability in traits, measured as SD, was negatively correlated with complexity and coral cover for all cases where significant relationships were detected (S4 Table). The first two PCoA axes were able to explain a substantial amount of the variation (axis 1 ~33%, axis 2 ~16%) of traits among species (Fig 2A), while the inclusion of axis 3 and 4 resulted in ~68% explanation of the variation (axis 3 ~10%, axis 4 ~9%). T_H_ and HR_S_ contributed to the separation of ten out of fifteen of the most abundant species in the PCoA (Fig 3B). Of the most abundant species, nine belonged to the Pomacentridae family, four to Labridae, one to Labridae (tribe: Scarini) and one to Balistidae (Fig 2A).

**Table 2:** CWM trait-environment relationships. The table lists significant results of linear regression of individual transect-level CWM of the examined traits against three environmental parameters (rugosity, coral cover and distance from mainland).

	Rugosity				Live Coral				Distance			
Trait	Coef.	Inter.	*F*	*r*^2^	Coef.	Inter.	*F*	*r*^2^	Coef.	Inter.	*F*	*r*^*2*^
DR	-0.066	0.904	0.02	0.00	-0.006	0.900	0.90	0.05	-0.006	0.920	1.23	0.06
L_m_	-1.168	2.625	7.44	**0.29**	-0.014	0.903	6.81	**0.27**	-0.008	0.793	2.25	0.11
DG_P_	-0.001	0.006	0.02	0.00	-0.001	0.006	1.79	0.09	-0.001	0.006	2.53	0.12
DG_C1_	-0.017	0.041	8.12	**0.31**	-0.001	0.016	11.13	**0.38**	-0.001	0.016	8.59	**0.32**
DG_C2_	-0.009	0.037	0.19	0.01	-0.001	0.027	1.25	0.06	-0.001	0.027	1.13	0.06
DG_Z_	-0.004	0.033	0.02	0.00	-0.001	0.037	2.45	0.12	-0.001	0.033	0.96	0.05
DG_H1_	-0.006	0.019	1.30	0.06	-0.001	0.010	1.57	0.08	-0.001	0.009	0.35	0.02
DG_H2_	0.011	0.008	0.15	0.01	-0.001	0.042	5.48	**0.22**	-0.001	0.030	0.14	0.01
DG_Co_	0.001	0.007	0.01	0.00	-0.001	0.009	1.42	0.07	-0.001	0.008	0.16	0.01
DG_D_	-0.010	0.030	1.37	0.07	-0.001	0.018	7.32	**0.28**	-0.001	0.016	1.39	0.07
HR_S_	-0.046	0.104	12.12	**0.40**	-0.001	0.036	11.39	**0.39**	-0.001	0.031	3.33	0.16
HR_M_	-0.017	0.043	4.08	0.18	-0.0003	0.020	7.02	**0.28**	-0.001	0.018	3.71	0.17
HR_L_	-0.024	0.057	4.34	**0.19**	-0.001	0.022	4.70	**0.21**	-0.001	0.020	2.18	0.11
SB_A_	0.001	0.025	0.01	0.00	-0.001	0.032	1.61	0.08	-0.001	0.029	0.32	0.02
SB_B_	-0.005	0.013	1.44	0.07	-0.001	0.006	0.24	0.01	-0.001	0.005	0.01	0.00
SB_C_	0.003	0.019	0.02	0.00	0.001	0.024	0.01	0.00	-0.001	0.028	0.42	0.02
SB_D_	0.004	0.001	0.20	0.01	-0.001	0.011	1.46	0.07	-0.001	0.010	1.00	0.05
SB_E_	0.001	0.008	0.13	0.00	-0.001	0.013	4.81	**0.21**	-0.001	0.010	0.02	0.00
T_H_	-0.005	0.026	1.95	0.01	-0.001	0.031	16.44	**0.49**	-0.001	0.027	6.06	**0.26**
T_L_	-0.005	0.014	1.85	0.07	-0.001	0.008	5.13	**0.22**	-0.001	0.007	1.03	0.05
T_M_	-0.004	0.023	1.13	0.01	-0.001	0.028	12.87	**0.43**	-0.001	0.025	5.49	**0.24**

Coefficients (Coef.), the y-intercept (Inter.), the *F*_(1,19)_ ratio and the strength of the relationship (*R*^2^) are provided. Significant relationships (*p*<0.05) are indicated by bold *r*^2^ values.

Based on analysis of the mean index values (Fig 3), we found little evidence that live coral cover, rugosity or distance from the mainland affected functional diversity and richness at a constant rate. For SESFRic, no sites differed from the values expected in randomly-assembled communities, while SESRaoQ at BL was below the expected values (Fig 3).

**Fig 4:**
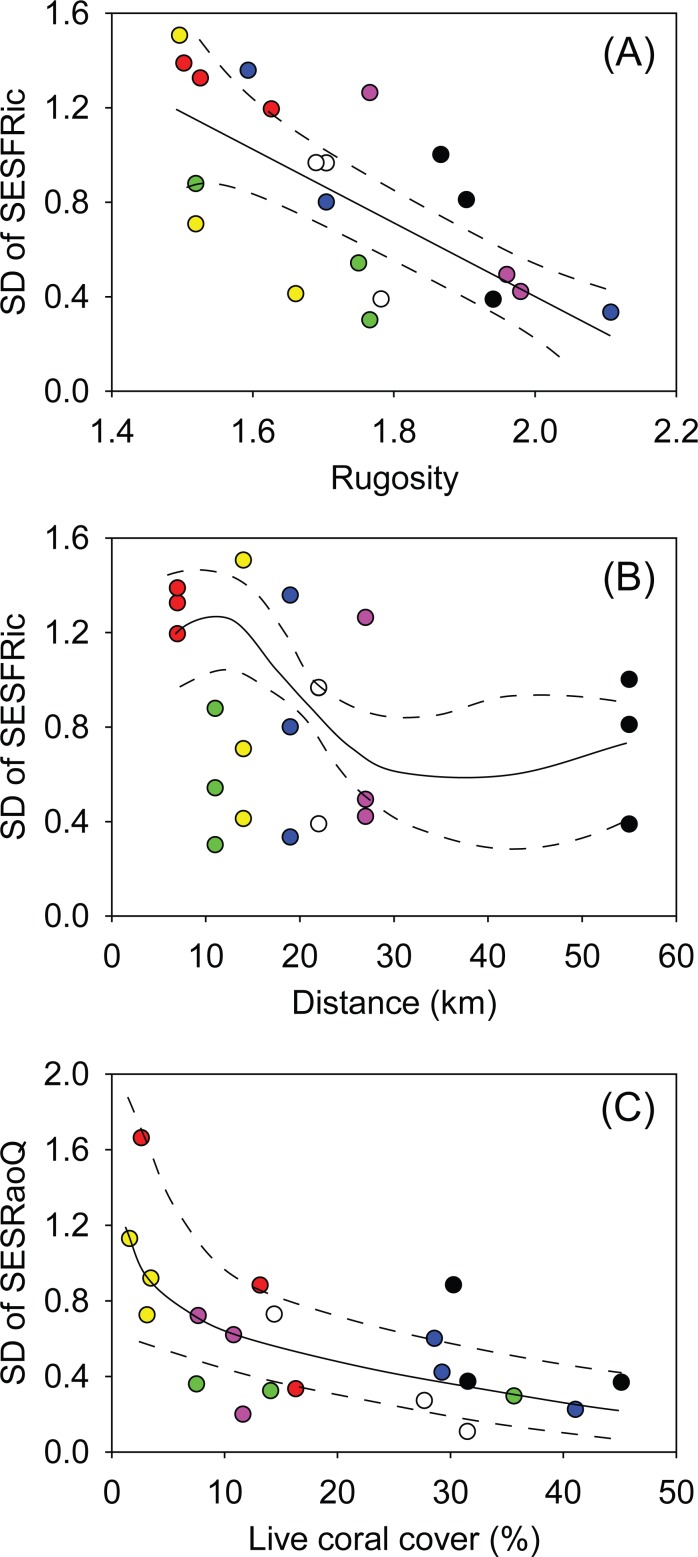
Variability (standard deviation, SD) in the standard effect size of FRic (SESFRic) and RaoQ (SESRaoQ) as a function of significant environmental covariates. The GAM for SESFRic indicated significant relationship with A) rugosity and B) distance from shore and a GLM indicated that C) coral cover was significantly related with SESRaoQ. Plots include the expected (solid line) and ±95% confidence intervals (dashed lines). Colours represent sampling sites and correlate with Fig 1: red = SA, green = BL, yellow = BO, blue = BA, white = LU, pink = KA, and black = KP.

Model validation with consideration of AICs indicated that a GAM model including the covariates rugosity and distance from shore was most suited for evaluating the SD of SESFRic (S2 Table). For SESRaoQ, a GLM with the covariate coral cover was selected (S3 Table). Although the mean values of the two indices did not directly relate to the environmental variables, within-transect variability displayed strong correlations (Fig 4). SD of SESFRic showed a significant relationship with rugosity (GAM, *df* = 1, *F* = 23.8, *p* < 0.001; Fig 4A and S2 Table) and with distance from shore (GAM, *df* = 4, *F* = 3.6, *p* = 0.027; Fig 4B and S2 Table). For SD of SESFRic, the inclusion of both rugosity and distance from shore explained 76.3% of the deviance in the GAM. Variability in SESFRic decreased linearly with an increase in rugosity. Variability in SESFRic decreased sharply within the first 25 km from shore but remained similar beyond that distance. However, a lack of sampling sites between 27 km and 55 km distance reduced the accuracy of the GAM within this range. SD of SESRaoQ showed a strong, significant relationship with coral cover (GLM, *F*_19,20_, *p* = 0.002; Fig 4C), with coral cover explaining approximately 40.2% of the deviance. Variability in SESRaoQ decreased considerably with an increase in coral cover. For instance, a coral cover in the range of 0–10% had twice higher SD of SESRaoQ than when coral cover was in the range of 30–40% (Fig 4C).

When individual traits were dropped for the sensitivity analysis, T-tests failed to identify subsets of traits that differed significantly from the observed data suggesting that no individual trait had a disproportionally high influence on the original model (S1 Fig). When the weights of the traits were removed, the relationships between SD of SESFRic with rugosity and distance mirrored results of the weighted traits (S2 Fig). These results were not completely replicated for the unweighted SD of SESRaoQ and live coral cover (S2 Fig). Here, one transect each at SA, BA and KP increased in variability suggesting that the selection of weighting of traits has some influence on the model output for SESRaoQ.

## Discussion

Coral reefs around the world are facing multiple disturbances, both local and global, which are increasingly understood as having impacts not only on biodiversity, but also on the functional composition of the communities [65–68]. We used a trait-based approach to investigate the functional assembly of coral reef fish communities along an environmental gradient. Most previous studies considering functional composition have focused on the change in mean values of functional indices and/or mean species trait diversity values [22–24], however we have also considered variability around repeated samples to account for short-term variation within fish communities that occur under increasing habitat heterogeneity [9,69].

Mean functional indicators of the fish communities did not display a strong, correlative relationship with any of the investigated environmental parameters. Conversely, the variability of fish communities, across samples repeated on a small temporal scale (a few hours), decreased with increases in coral cover, rugosity and distance from the mainland, thus indicating that the state of the coral reef habitat is important in functional constancy of the fish assemblage. There was a stronger relationship between fish community variability and the environment with regards to functional richness (SESFRic) than diversity (SESRaoQ) indicating that total trait space (functional richness) became more variable with greater impact than the proportional balance of abundance-weighted functional composition (functional diversity). It should be noted, however, that patterns seen in functional diversity (SD of SESRaoQ) are affected by the weighting of traits where our choices resulted in reduced variability among transects (Fig 4 and S2 Fig).

Studies of taxonomic composition of fish communities subject to habitat degradation have demonstrated increased variability in parameters of biodiversity of reef fish communities exposed to habitat loss through coral mining [32]. Non-mined sites displayed much higher abundances of small planktivores (pomacentrids), suggesting an impact on trophic functioning within mined sites. Similarly, Pratchett et al. [70] and Alvarez-Filip et al. [71] observed that the loss of structural complexity and live coral cover led to a disproportional impact on smaller-bodied species at lower trophic levels and species depending on live corals for food and shelter.

When considering specific traits, our results help explain mechanisms that result in increased variability of taxonomic and functional composition. The CWM of maximum length (L_M_) showed the steepest decline in response to both structural complexity and coral cover and communities at sites with reduced coral cover comprised relatively more individuals with large (HR_W_) and medium (HR_M_) home range sizes. The only trait CWM responding negatively to all three independent environmental variables was consumption of small invertebrates (DG_C1_), which was characteristic of several larger labrid species that displayed high relative abundances when live coral-associated damselfishes became scarce. While change in home ranges reveal the attributes that increase turnover between repeated samples, contributing to the observed variability at degraded sites, it is the feeding traits that identify a more detailed response of communities.

The higher CWM values of detritivory and microalgae/cyanobacteria feeding at sites with lower coral cover reflect shifts in benthic resources, with detritus, microalgae and cyanobacteria being inversely related to live coral (Plass-Johnson et al. unpublished manuscript). In Spermonde, these trophic niches were targeted by several pomacentrid species that were highly abundant at the degraded sites, have small home ranges (HR_S_), feed on micro-algae (DG_H2_), and/or have high reproductive turnover (T_H_) (i.e. *Pomacentrus aurifrons*, *Pomacentrus adelus* or *Pomacentrus alexanderae*). The observed trait responses suggest that degraded sites allowed a few species with a trophic dependence on the replaced habitat (i.e. micro-algae) to become proportionally more abundant at some transects, leading to fewer species and high turnover on short temporal scales. Furthermore, this may help explain why, in contrast to the initial prediction based on Pratchett et al. [70] and Alvarez-Filip et al. [71], small home range size was negatively correlated with coral cover and structural complexity. The higher coral cover and rugosity of off-shore sites were more adequate for a broader range of species, allowing individuals of more species to co-exist, and contributing to a more stable community composition.

With respect to individual traits, it is important to note that variability increased with decreasing habitat quality (S4 Table), similarly to the composite indicators FRic and RaoQ. It is this variability of specific traits, resulting in differing multidimensional trait space that affected FRic so strongly.

Multiple studies have applied trait-based functional indices to identify community assembly rules [13,16,18,19,57,58]. Unlike these other studies, the Spermonde Archipelago did not display clear patterns based on mean index values, indicating changes in the environment did not completely remove traits from the communities. However, mechanisms structuring coral reef fish assemblages of the Spermonde Archipelago were more apparent in the variability of functional diversity indices. Responses in the CWM of particular traits suggest a close relationship between these traits and increasing stochasticity in fish community functioning in relation to increased disturbance. For coral reef fishes, habitat filtering initially would act upon traits resulting in altered abundances. While this would not completely remove these traits from the communities, altered abundances would change the proportional occurrence of traits contributing to increased stochasticity of functional indices evident in repeated samples. For example, localised disturbances may lead to loss of corals and structural complexity, resulting in homogenisation of habitat at the within-transect scale, and thus leading to more weight on medium-range, less site-attached species [71]. Importantly, some traits showed responses that were in contrast with previous observations (i.e. small home range size), but were generally in line with habitat filtering allowing particular traits to become abundant. Processes that lead to more heterogeneity in the occurrence of traits without completely removing them from a site would not become apparent in the means of the indices, as variability becomes masked when averaging across repeated transects. Increased variability without simultaneous changes in mean indices may thus constitute a useful early warning sign of habitat degradation.

The present results underline that, within the Spermonde Archipelago, there are linkages between the functional assembly of coral reef fishes, their traits (particularly those traits related to mobility), and an environmental gradient of increased habitat degradation. In light of increasing localised stressors on reefs around the world, this is of high concern because feedback loops between reef fishes and the environment [72,73] are key in the maintenance of coral reef ecosystem functioning [72]. In the Spermonde Archipelago, increases in the variability of functional diversity and richness were associated with a more impacted habitat, suggesting that increased variability in the functional composition is a symptom of a change in species’ interactions. Disturbance may have a greater impact on the most variable reefs because they may lack the capacity to respond consistently to stressors, and thus may comprise less response diversity [74,75] compared to reefs of lower variability.

It should be kept in mind that limitations exist within our experimental design that impede some ecological interpretation. Firstly, using diver-based underwater visual surveys can lead to results biased by the behaviour of fishes [76,77]. Care was taken to minimise these biases, e.g. by using fixed-distance transects and waiting 30 min after deployment of transect tapes before resuming fish counts [76]. Next, spearfishing is not a major activity in the study area, and all sites were open to fishing, reducing potential fishing-induced behavioural responses of fishes to divers [77]. However, there remains the possibility of some fish species not adversely affected by divers to be over-represented within the data set. More information on species-specific responses to the presence of divers is needed to account for potential methodological biases, and additional methods (e.g. remote video surveys) should be applied to study variability in fish communities. Thirdly, all analyses based on the species-specific trait composition of a community will come with inherent subjectivity because we are unable to measure all characteristics of a species that represent traits. Thus, our study probably underestimates biological variation within the fish communities. Nonetheless, we believe that our chosen traits are broad proxies for general ecological interactions of the species. These traits have been used in previous studies and they are also easily accessible for future comparisons. Lastly, this study should be replicated in other coral reef systems to identify true generalities of the ecosystem patterns seen in this study. Nonetheless, reasonably wide spatial replication throughout the Spermonde Archipelago added to the understanding of functional variability and competitive interactions along disturbance gradients, thus contributing to improving our predictions of how coral reefs will react in a changing environment [8]. Based on these findings, we suggest that further assessments of variability of functional indices in response to stress are a promising avenue for future work on community assembly and functioning under conditions of environmental change. Of particular interest are the mechanisms underlying the observed response in variability to habitat degradation. The present study could only provide some indications in this respect. We suggest that future studies address the roles of species’ home range sizes, resource specialisation, and multiple spatial scales of habitat heterogeneity in contributing to the observed link between community variability and habitat degradation.

## Supporting Information

S1 Fig Sensitivity analysis. Each index (A: FRic and B: RaoQ) was calculated for all combinations of 5 of 6 weighted traits. This was also combined with null models based on 999 iterations, calculating SES values as stated in the primary paper (Measuring observed and expected functional diversity). Means (+/-SE) included all sites and replicates. Value of the observed model (full set of 6 traits) is indicates in black and reduced models are in gray. The label below the reduced models indicates the trait that was dropped. T-tests failed to identify subsets of traits that differed significantly from the observed data suggesting that no individual trait had disproportionally high influence on the primary model.
(DOCX)

S2 Fig Relationship (standard deviation, SD) in the unweighted standard effect size of FRic (SESFRic) and RaoQ (SESRaoQ) as a function of environmental covariates. These figures are provided as visual comparison with the weighted results (Fig 3). Similar trends in SESFRic can be seen between the weighted and weighted SDs with rugosity and distance, however the relationship is not as clear for SESRaoQ and live coral cover. GAM for the relationship between the unweighted SD of SESFRic and distance (GAM; Ref. F = 6.794. Dev. Exp. = 6.94%, AIC = 30.17, Int. *p* < 0.001. Factor *p* = 0.01) and rugosity (GAM; Ref. F = 8.097. Dev. Exp. = 38.0%, AIC = 25.17, Int. *p* < 0.001. Factor *p* = 0.01) were similar to the weighted results. GLM results for the relationhip between SD of SESRaoQ and live coral was not significant (GLM, Resid. Dev. = 8.932, R^2^ = 0.09, AIC = 8.921, Int.*p* = 0.76, F1*p* = 0.45).
(DOCX)

S3 Fig Correlation coefficients among all environmental covariates used as predictors for the SD of SESFRic and SESRaoQ. A correlation coefficient of 0.8 was considered a strong relationship at which a covariate would be removed from the subsequent models. Colours represent sampling sites and correspond to Fig 1: red = SA, green = BL, yellow = BO, blue = BA, white = LU, pink = KA, and black = KP.
(DOCX)

S1 Table Average number of functional groups, average species richness, and average abundance of individual fishes (± SE 250m^-2^) for each of the surveyed Spermonde islands. Sites are listed from left to right in order of increasing distance from Makassar.
(DOCX)

S2 Table AIC scores of competing GAMs assessing their relationship with FRic. (DOCX)

S3 Table AIC scores of competing GLMs assessing their relationship with RaoQ. Models are presented based on increasing AIC score. *R*^2^ was calculated by subtracting the residual deviance from the null deviance and then diving by the null deviance. F1, F2, F3 correspond to the model’s factors (Factor).
(DOCX)

S4 Table CWM trait-environment relationships. The table lists significant results of linear regression of transect-level standard deviations around the mean for individual CWM traits against three environmental parameters (rugosity, coral cover and distance from mainland).
(DOCX)
