# Supplementary material for: Non-Random Variability in Functional Composition of Coral Reef Fish Communities along an Environmental Gradient
Source: PLoS One. 2016 Apr 21;11(4):e0154014. doi: 10.1371/journal.pone.0154014 (PMC4839599; doi:10.1371/journal.pone.0154014)
Supplement: S1 Fig — Each index (A: FRic and B: RaoQ) was calculated for all combinations of 5 of 6 weighted traits. This was also combined with null models based on 999 iterations, calculating SES values as stated in the primary paper (Measuring observed and expected functional diversity). Means (+/-SE) included all sites and replicates. Value of the observed model (full set of 6 traits) is indicates in black and reduced models are in gray. The label below the reduced models indicates the trait that was dropped. T-tests failed to identify subsets of traits that differed significantly from the observed data suggesting that no individual trait had disproportionally high influence on the primary model. (DOCX) [file pone.0154014.s001.docx]

S1 Fig. Sensitivity analysis. Each index (A: FRic and B: RaoQ) was calculated for all combinations of 5 of 6 weighted traits. This was also combined with null models based on 999 iterations, calculating SES values as stated in the primary paper (Measuring observed and expected functional diversity). Means (+/-SE) included all sites and replicates. Value of the observed model (full set of 6 traits) is indicates in black and reduced models are in gray. The label below the reduced models indicates the trait that was dropped. T-tests failed to identify subsets of traits that differed significantly from the observed data suggesting that no individual trait had disproportionally high influence on the primary model.
