# Supplementary material for: Non-Random Variability in Functional Composition of Coral Reef Fish Communities along an Environmental Gradient
Source: PLoS One. 2016 Apr 21;11(4):e0154014. doi: 10.1371/journal.pone.0154014 (PMC4839599; doi:10.1371/journal.pone.0154014)
Supplement: S2 Fig — These figures are provided as visual comparison with the weighted results (Fig 3). Similar trends in SESFRic can be seen between the weighted and weighted SDs with rugosity and distance, however the relationship is not as clear for SESRaoQ and live coral cover. GAM for the relationship between the unweighted SD of SESFRic and distance (GAM; Ref. F = 6.794. Dev. Exp. = 6.94%, AIC = 30.17, Int. p < 0.001. Factor p = 0.01) and rugosity (GAM; Ref. F = 8.097. Dev. Exp. = 38.0%, AIC = 25.17, Int. p < 0.001. Factor p = 0.01) were similar to the weighted results. GLM results for the relationhip between SD of SESRaoQ and live coral was not significant (GLM, Resid. Dev. = 8.932, R2 = 0.09, AIC = 8.921, Int.p = 0.76, F1p = 0.45). (DOCX) [file pone.0154014.s002.docx]

**S2 Fig. Relationship (standard deviation, SD) in the unweighted standard effect size of FRic (SESFRic) and RaoQ (SESRaoQ) as a function of environmental covariates.** These figures are provided as visual comparison with the weighted results (Fig. 3). Similar trends in SESFRic can be seen between the weighted and weighted SDs with rugosity and distance, however the relationship is not as clear for SESRaoQ and live coral cover. GAM for the relationship between the unweighted SD of SESFRic and distance (GAM; Ref. F = 6.794. Dev. Exp. = 6.94%, AIC = 30.17, Int. *p* < 0.001. Factor *p* = 0.01) and rugosity (GAM; Ref. F = 8.097. Dev. Exp. = 38.0%, AIC = 25.17, Int. *p* < 0.001. Factor *p* = 0.01) were similar to the weighted results. GLM results for the relationhip between SD of SESRaoQ and live coral was not significant (GLM, Resid. Dev. = 8.932, R^2^ = 0.09, AIC = 8.921, Int.*p* = 0.76, F1*p* = 0.45).
