# Supplementary material for: Non-Random Variability in Functional Composition of Coral Reef Fish Communities along an Environmental Gradient
Source: PLoS One. 2016 Apr 21;11(4):e0154014. doi: 10.1371/journal.pone.0154014 (PMC4839599; doi:10.1371/journal.pone.0154014)
Supplement: S3 Fig — A correlation coefficient of 0.8 was considered a strong relationship at which a covariate would be removed from the subsequent models. Colours represent sampling sites and correspond to Fig 1: red = SA, green = BL, yellow = BO, blue = BA, white = LU, pink = KA, and black = KP. (DOCX) [file pone.0154014.s003.docx]

S3 Fig. Correlation coefficients among all environmental covariates used as predictors for the SD of SESFRic and SESRaoQ. A correlation coefficient of 0.8 was considered a strong relationship at which a covariate would be removed from the subsequent models. Colours represent sampling sites and correspond to Fig.1: red = SA, green = BL, yellow = BO, blue = BA, white = LU, pink = KA, and black = KP.
