# Supplementary material for: Non-Random Variability in Functional Composition of Coral Reef Fish Communities along an Environmental Gradient
Source: PLoS One. 2016 Apr 21;11(4):e0154014. doi: 10.1371/journal.pone.0154014 (PMC4839599; doi:10.1371/journal.pone.0154014)
Supplement: S1 Table — Sites are listed from left to right in order of increasing distance from Makassar. (DOCX) [file pone.0154014.s004.docx]

S1 Table. Average number of functional groups, average species richness, and average abundance of individual fishes (± SE 250m^-2^) for each of the surveyed Spermonde islands. Sites are listed from left to right in order of increasing distance from Makassar.

|  | SA | BL | BO | BA | LU | KA | KP |
| --- | --- | --- | --- | --- | --- | --- | --- |
| Distance (km) | 7 | 11 | 14 | 19 | 22 | 27 | 55 |
| Species richness | 22.3 | 17.6 | 15.4 | 31.3 | 25.8 | 29.6 | 29.0 |
| (SE) | (1.92) | (1.39) | (2.10) | (1.22) | (1.38) | (1.44) | (1.71) |
| Abundance | 267.4 | 147.2 | 335.7 | 1243.3 | 426.7 | 666.4 | 438.2 |
| (SE) | (20.3) | (19.1) | (25.5) | (327.2) | (45.2) | (115.5) | (60.7) |
