# Supplementary material for: Non-Random Variability in Functional Composition of Coral Reef Fish Communities along an Environmental Gradient
Source: PLoS One. 2016 Apr 21;11(4):e0154014. doi: 10.1371/journal.pone.0154014 (PMC4839599; doi:10.1371/journal.pone.0154014)
Supplement: S2 Table — (DOCX) [file pone.0154014.s005.docx]

**S2 Table. AIC scores of competing GAMs assessing their relationship with FRic**

| Factor | Ref. df | *df* | F | Dev. Exp. | AIC | Int. | F1 | F2 | F3 |
| --- | --- | --- | --- | --- | --- | --- | --- | --- | --- |
| Dist +Rug | 3 | 3 | 5.75 | 71.9 | 2.878 | **<0.001** | **0.007** | **<0.001** |  |
| LC+Rug+Dist | 3 | 3 | 1.141 | 77.6 | 4.131 | **<0.001** | 0.369 | **<0.001** | 0.241 |
| LC+Rug | 3 | 3 | 1.569 | 54.1 | 13.367 | **0.022** | 0.236 | **0.003** |  |
| Rug | 2 | 2 | 6.253 | 42.7 | 14.107 | **<0.001** | **0.009** |  |  |
| LC | 3 | 3 | 1.142 | 16.5 | 24.247 | **<0.001** | 0.36 |  |  |
| Dist | 2 | 2 | 0.908 | 7.93 | 24.372 | **<0.001** | 0.421 |  |  |
| LC+Dist | 3 | 3 | 0.833 | 23.1 | 28.461 | **<0.001** | 0.714 | 0.498 |  |
