# Supplementary material for: Non-Random Variability in Functional Composition of Coral Reef Fish Communities along an Environmental Gradient
Source: PLoS One. 2016 Apr 21;11(4):e0154014. doi: 10.1371/journal.pone.0154014 (PMC4839599; doi:10.1371/journal.pone.0154014)
Supplement: S3 Table — Models are presented based on increasing AIC score. R2 was calculated by subtracting the residual deviance from the null deviance and then diving by the null deviance. F1, F2, F3 correspond to the model’s factors (Factor). (DOCX) [file pone.0154014.s006.docx]

**S3 Table. AIC scores of competing GLMs assessing their relationship with RaoQ.** Models are presented based on increasing AIC score. *R*^2^ was calculated by subtracting the residual deviance from the null deviance and then diving by the null deviance. F1, F2, F3 correspond to the model’s factors (Factor).

| Factor | Null dev. | *df* | Resid. Dev. | *df* | *R*^2^ | AIC | Int. | F1 | F2 | F3 |
| --- | --- | --- | --- | --- | --- | --- | --- | --- | --- | --- |
| LC | 8.249 | 20 | 5.096 | 19 | 0.38 | 5.44 | **0.002** | **0.002** |  |  |
| LC+Dist | 8.249 | 20 | 4.851 | 18 | 0.41 | 6.36 | **0.003** | **0.005** | 0.325 |  |
| LC+Rug | 8.249 | 20 | 5.069 | 18 | 0.39 | 7.32 | 0.431 | **0.008** | 0.752 |  |
| LC+Rug+Dist | 8.249 | 20 | 4.825 | 17 | 0.42 | 8.24 | 0.833 | **0.007** | 0.755 | 0.338 |
| Rug | 8.249 | 20 | 7.723 | 19 | 0.06 | 14.60 | 0.654 | 0.252 |  |  |
| Dist | 8.249 | 20 | 7.951 | 19 | 0.04 | 15.25 | **0.004** | 0.418 |  |  |
| Rug+Dist | 8.249 | 20 | 7.713 | 18 | 0.07 | 16.57 | 0.786 | 0.457 | 0.877 |  |
