# Supplementary material for: Non-Random Variability in Functional Composition of Coral Reef Fish Communities along an Environmental Gradient
Source: PLoS One. 2016 Apr 21;11(4):e0154014. doi: 10.1371/journal.pone.0154014 (PMC4839599; doi:10.1371/journal.pone.0154014)
Supplement: S4 Table — The table lists significant results of linear regression of transect-level standard deviations around the mean for individual CWM traits against three environmental parameters (rugosity, coral cover and distance from mainland). (DOCX) [file pone.0154014.s007.docx]

**S4 Table. CWM trait-environment relationships.** The table lists significant results of linear regression of transect-level standard deviations around the mean for individual CWM traits against three environmental parameters (rugosity, coral cover and distance from mainland).

| Trait | Slope | y-int | *R*^2^ | *P*-value |
| --- | --- | --- | --- | --- |
| *Rugosity* |  |  |  |  |
| L_M_ | -1.1675 | 2.6251 | 0.29 | 0.014 |
| DG_C1_ | -0.0172 | 0.0409 | 0.31 | 0.011 |
| HR_S_ | -0.0461 | 0.1035 | 0.40 | 0.002 |
| HR_M_ | -0.0165 | 0.0423 | 0.21 | 0.049 |
| T_H_ | -0.0409 | 0.0885 | 0.52 | 0.001 |
|  |  |  |  |  |
| *Coral Cover* |  |  |  |  |
| L_M_ | -0.0173 | 1.0068 | 0.29 | 0.012 |
| DG_C1_ | -0.0002 | 0.0158 | 0.38 | 0.004 |
| DG_Z_ | -0.0004 | 0.0274 | 0.27 | 0.026 |
| DG_H2_ | -0.0009 | 0.0469 | 0.35 | 0.006 |
| DG_D_ | -0.0003 | 0.0181 | 0.28 | 0.014 |
| HR_S_ | -0.0008 | 0.0382 | 0.58 | <0.001 |
| HR_M_ | -0.0003 | 0.0197 | 0.28 | 0.016 |
| HR_L_ | -0.0004 | 0.0232 | 0.24 | 0.025 |
| T_H_ | -0.0005 | 0.0309 | 0.49 | <0.001 |
|  |  |  |  |  |
| *Distance* |  |  |  |  |
| L_M_ | -0.0281 | 1.1485 | 0.25 | 0.049 |
| DG_C1_ | -0.0004 | 0.0187 | 0.25 | 0.049 |
| HR_S_ | -0.0010 | 0.0409 | 0.26 | 0.035 |
| HR_M_ | -0.0006 | 0.0240 | 0.27 | 0.034 |
| SB_D_ | -0.0004 | 0.0138 | 0.29 | 0.024 |
| T_L_ | -0.0002 | 0.0093 | 0.48 | 0.003 |
